# Case of Phthisis Pulmonalis

**Published:** 1805-04-01

**Authors:** 


### *To the Editors of the Medical and Physical Journal.*

Gentlemen,

Being one of the medical profession, I think it my duty to communicate every thing which has a tendency to increase the knowledge and cure of diseases. The following case of Phthisis Pulmonalis, although but short, I hope will not be unacceptable to the readers of your excellent Journal.

A gentleman in the west of England, after being under the care of an eminent medical practitioner for two months, applied to a friend of mine; and on examination found him to be in the last stage of a consumption. The symptoms were, a frequent cough, a copious and fœtid purulent expectoration with two daily exacerbations, which were preceded by a sense of coldness; his urine was high coloured, and deposited a copious branny red sediment; appetite not much impaired, thirst inconsiderable. He was ordered the following emetic draught. ℞. Cupri vitriolati gr. x. aquæ puræ ℥ij. M. vespere sumend. After the emetic had operated he took an anodyne draught, cum tinct. op. gt. xx. He slept much better after the anodyne draught than he had for some time before; he then was ordered the digitalis with opium in the following manner. ℞. Pulv. digital. opii purif. aa. gr. x. Cons. ros. q. s. ut fiat pilulæ xx. Sumat j. nocte maneque. He also took the following decoction. ℞. Lichen. islandic. ℥j. rad. glyc. inc. ʒij. aquæ puræ ℔jß. coque ad ℔j. et cola sumat ℥iv. ter quaterve in die.

After taking these medicines three days the hectic symptoms left him, and he was able to take an airing on horseback, which he had not done for some time before; and by strictly following the above medicines eight weeks, he perfectly recovered. His diet consisted of shell fish, fowl, and vegetables

I am, &c.

*Bristol, Feb.* 10, 1805. W. L.

